# Evaluation of Thrombomodulin, Heart-Type Fatty-Acid-Binding Protein, Pentraxin-3 and Galectin-3 Levels in Patients with Myocardial Infarction, with and Without ST Segment Elevation

**DOI:** 10.3390/jcm14031015

**Published:** 2025-02-05

**Authors:** Naile Fevziye Misirlioglu, Gulbahar Guler Orucoglu, Burcu Bıcakhan, Suat Hayri Kucuk, Solen Himmetoglu, Sena Baykara Sayili, Gulenay Defne Ozen, Hafize Uzun

**Affiliations:** 1 Department of Biochemistry, Gaziosmanpaşa Training and Research Hospital, University of Health Sciences, 34098 Istanbul, Turkey; 2 Department of Emergency, Gaziosmanpaşa Training and Research Hospital, University of Health Sciences, 34098 Istanbul, Turkey; gulbahar.91.gg@gmail.com; 3 Department of Cardiovascular Surgery, Gaziosmanpaşa Training and Research Hospital, University of Health Sciences, 34098 Istanbul, Turkey; isiksungur@gmail.com; 4 Department of Biochemistry, Istanbul Physical Medicine and Rehabilitation Training and Research Hospital, University of Health Sciences, 34186 Istanbul, Turkey; suatkucuk@gmail.com; 5 Department of Medical Biochemistry, Faculty of Medicine, Biruni University, 34015 Istanbul, Turkey; shimmetoglu@biruni.edu.tr; 6 Biruni University Research Center (B@MER), Biruni University, 34015 Istanbul, Turkey; 7 Department of Emergency Medicine, Istanbul Training and Research Hospital, 34098 Istanbul, Turkey; senabaykara@gmail.com; 8 Department of Psychology, McGill University, Montreal, QC H3A 1G1, Canada; daphne.oz@mail.mcgill.ca; 9 Department of Medical Biochemistry, Faculty of Medicine, Istanbul Atlas University, 34403 Istanbul, Turkey; huzun59@hotmail.com

**Keywords:** myocardial infarction, STEMI, NSTEMI, thrombomodulin, heart-type fatty-acid-binding protein, pentraxin-3, galectin-3

## Abstract

**Background:** Medical history, ECG findings and cardiac markers are used in the diagnosis of myocardial infarction (MI). Biomarkers used especially for the diagnosis of MI include high-sensitivity troponins (hsTns), creatine kinase-MB (CK-MB), lactate dehydrogenase (LDH), myoglobin, cardiac myosin-binding protein C and new cardiac biomarkers. This study evaluated the levels of serum thrombomodulin (TM), heart-type fatty-acid-binding protein (H-FABP), pentraxin-3 (PTX-3) and galectin-3 (Gal-3) to determine their utility in distinguishing between ST-elevation myocardial infarction (STEMI) and non-ST-elevation myocardial infarction (NSTEMI). **Methods:** This study included a total of 180 patients (90 patients with acute STEMI and 90 patients with NSTEMI) who presented to the Gaziosmanpaşa Training and Research Hospital, Cardiovascular Surgery and Emergency Department, with ischemic chest pain lasting longer than 30 min. Ninety healthy volunteers were included as the control group. **Results:** Serum levels of *N*-terminal pro-brain natriuretic peptide (NT-proBNP), TM, H-FABP, PTX-3 and Gal-3 were significantly different across the STEMI, NSTEMI and control groups (*p* < 0.001). Strong positive correlations were observed between NT-proBNP and TM, H-FABP, PTX-3 and Gal-3 in the STEMI group. ROC analysis demonstrated excellent diagnostic accuracy for these biomarkers in distinguishing STEMI from NSTEMI and control groups. **Conclusions:** Vascular inflammation plays an important role in the pathophysiology of STEMI and NSTEMI. A comprehensive cardiac biomarker panel enhances diagnostic accuracy and risk stratification, particularly when distinguishing between STEMI and NSTEMI. The biomarkers hs-TnI, CK-MB, NT-proBNP, TM, H-FABP, PTX-3 and Gal-3 offer complementary information when used together as a panel. Further research and validation are essential to establish standardized protocols for their widespread use.

## 1. Introduction

Myocardial infarction (MI) is defined as acute myocardial injury detected by cardiac biomarkers in the presence of clinical evidence of acute myocardial ischemia. The main pathophysiologic cause of acute coronary syndrome (ACS) is a disturbance in the balance between oxygen supply and demand of myocardial tissue [1]. The most common cause of ACS is plaque rupture leading to thrombotic vessel occlusion. ACS includes a range of conditions, and patients with these those with recent changes in clinical symptoms or signs, with or without changes in 12-lead electrocardiogram (ECG) and acute elevation of cardiac troponin (cTn) concentrations. ACS is divided into four groups: ST-elevation myocardial infarction (STEMI), non-ST-elevation myocardial infarction (NSTEMI), patients with myocardial damage proven by elevated markers of damage (NSTEMI) and patients with unstable angina [2].

Although cardiac-specific biomarkers including natriuretic peptides [atrial natriuretic peptide (ANP), brain natriuretic peptide (BNP)] and high-sensitivity troponin (hsTn)] are widely used in clinical practice, the utility of other biomarkers has yet to be proven [3,4,5,6]. It has been reported that the association of the activity level of the coagulation factor thrombomodulin (TM) may provide a new tool to assess the prognosis of acute MI [7,8,9].

A recently discovered, small, unbound cytoplasmic protein, and a potentially useful early biomarker, heart-type fatty-acid-binding protein (H-FABP), is present in high concentrations in the myocardial cell and is released into the circulation within minutes after myocardial ischemia [10]. H-FABP reflects sarcolemmal changes during acute myocardial ischemia. Its plasma kinetics and secretions are similar to myoglobin. Therefore, as a marker for early diagnosis of AMI (first two hours), it is evaluated [11]. There are a few studies on the use and value of H-FABP in the diagnosis of ACS [10,11,12,13,14].

Pentraxins (PTXs) are a group of proteins that regulate innate immunity in the body. These proteins, which include short and long pentraxins, have a cyclic multimeric structure. C-reactive protein (CRP) and serum amyloid P (SAP) are short PTXs. PTX-3 is recognized as a long PTX [15,16]. In a study conducted with patients hospitalized in a coronary care unit with ACS findings, plasma PTX-3 concentrations of the participants were found to be high. That study suggested that PTX-3 could be used as a marker in acute MI [17].

Galectin-3 (Gal-3) is a marker of organ fibrosis, including cardiac fibrosis [18]. Several clinical studies have shown that plasma levels of Gal-3 are associated with cardiac function and left ventricular filling pressures [19]. Gal-3 levels were found to be increased independently of MI severity and comorbidity indicators [20].

As mentioned above, cardiac biomarkers play an important role in the accurate, early diagnosis and treatment of ACS [21]. Biomarkers are seen as important tools for the detection of subclinical disease, diagnosis of acute or chronic coronary syndromes and risk stratification [22]. These biomarkers are of interest because they are involved in various aspects of myocardial injury, inflammation and cardiac remodeling, and may provide valuable diagnostic insights into the type and severity of AMI.

Serum levels of TM, H-FABP, PTX-3 and Gal-3 are associated with various aspects of cardiovascular injury and inflammation. These biomarkers could help improve the early detection and stratification of patients with ACS, providing a better understanding of their utility in clinical practice. This study focused on evaluating the levels of serum TM, H-FABP, PTX-3 and Gal-3 to determine their utility in distinguishing between STEMI and NSTEMI.

## 2. Materials and Methods

### 2.1. Ethical Considerations

This study was conducted according to the guidelines of the Declaration of Helsinki and approved by the Biruni University, Sciences Clinical Research Ethics Committee (file number of approval: 2024-BİAEK04-54; Date: 21 October 2024).

### 2.2. Subjects

The sample size was calculated using G*Power software (version 3.1.9.7) based on a medium effect size (f) of 0.25, alpha error of 0.05 and power (1-β) of 0.95 for comparing three groups; the minimum required total sample size was calculated to be 252 subjects (84 per group). To account for potential dropouts and missing data, we included 90 subjects per group (total *n* = 270), which provided adequate statistical power for detecting clinically meaningful differences in biomarker levels between STEMI, NSTEMI and control groups. This sample size also provided sufficient power for the correlation analyses and ROC curve analyses planned in this study.

This study included a total of 180 patients (90 patients with acute STEMI and 90 patients with NSTEMI) who presented to the Gaziosmanpaşa (GOP) Training and Research Hospital, Cardiovascular Surgery and Emergency Department, with ischemic chest pain lasting longer than 30 min. Patients presenting to the hospital with severe chest pain, signs of coronary stenosis and irregular heart contractions were evaluated for suspicious conditions by computed angiography. Blood samples were collected from 90 patients without occlusive lesions on coronary angiography and were included in the control group.

A detailed history and physical examination were performed in each patient. Twelve-lead ECGs were performed, and changes were recorded.

### 2.3. Inclusion Criteria

Patients were included based on the diagnostic criteria for NSTEMI and STEMI according to ESC/ACC guidelines [23]. Patients with ST elevation or new left bundle branch block on ECG were included in the STEMI group.

Healthy subjects aged ≥ 18 years and <75 years with no history of cardiovascular disease, peripheral vascular disease, cerebrovascular disease, malignancy, severe hepatic impairment and severe renal impairment were included.

### 2.4. Exclusion Criteria

Patients who presented after 12 h following chest pain, patients with chronic renal failure, patients with chronic muscle disease, patients with recent trauma or skeletal muscle injury, patients with heart failure and patients presenting with shock were excluded.

### 2.5. Blood Collection

Venous blood samples obtained from the participants were centrifuged at 4000 rpm for 5 min in the emergency laboratory of GOP Hospital, and all biochemical parameters were analyzed in serum samples on the same day. Serum samples for biochemical markers were stored at −80 °C until the study.

### 2.6. Assessment of Serum Thrombomodulin (TM) Levels

Serum TM levels were assessed using commercial ELISA assay (Human Human thrombomodulin ELISA Kit, Cat No: E1183Hu, BT LAB, Shanghai, China) according to the manufacturer. Standard curve range: 0.05–20 ng/mL; sensitivity: 0.021 ng/mL. Intra-assay: CV < 8%; inter-assay: CV < 10%.

### 2.7. Assessment of Serum Heart-Fatty-Acid-Binding Protein (H-FABP) Levels

Serum H-FABP levels were assessed using an ELISA Kit (Human-heart-fatty-acid-binding protein ELISA Kit, Cat No: E1213Hu, BT LAB, Shanghai, China) according to the instructions. Standard curve range: 0.05–18 ng/mL; sensitivity: 0.023 ng/mL. Intra-assay: CV < 8%; inter-assay: CV < 10%.

### 2.8. Measurement of Serum Pentraxin 3 (PTX-3) Levels

Serum PTX-3 levels were assessed using an ELISA Kit (Human Pentraxin 3 ELISA Kit, Cat No: E1938Hu, BT LAB, Shanghai, China). Standard curve range: 0.1–30 ng/mL; sensitivity: 0.05 ng/mL. Intra-assay: CV < 8%; inter-assay: CV < 10%.

### 2.9. Measurement of Serum Galectin-3 (Gal-3) Levels

Serum Gal-3 levels were assessed using an ELISA Kit (Human Galectin-3 ELISA Kit, Cat No: E1951Hu, BT LAB, Shanghai, China) according to the manufacturer. Standard curve range: 5–2000 pg/mL; sensitivity: 2.49 pg/mL. Intra-assay: CV < 8%; inter-assay: CV < 10%.

Routine biochemical parameters such as glucose, cholesterol and albumin in blood were measured with an automated analyzer (COBAS 8000, ROCHE-2007, Tokyo, Japan). Serum CRP levels were measured nephelometrically (Siemens-Dimention, Munich, Germany). The high-sensitive cTnI (hs-cTnI) levels were assessed by the immunofluorescent method using fluorescent antibody conjugates (ARCHITECTR, Abbott, TX, USA). The N-terminal proBNP (NT-proBNP) levels were assayed by the chemiluminescence method using an I2000 architect machine (ARCHITECTR, Abbott, TX, USA).

### 2.10. Statistical Analysis

All statistical analyses were performed using IBM SPSS Statistics version 21.0 (IBM Corp., Armonk, NY, USA). Visualizations of graphs were made with JASP software (version 0.17.1, Amsterdam, The Netherlands). Continuous variables were evaluated for normality using the Kolmogorov–Smirnov test. Variables with normal distribution were presented as mean ± standard deviation (SD), while non-normally distributed data were presented as median and interquartile range (IQR). Categorical variables were summarized as frequencies and percentages. For comparisons among the three groups (control, NSTEMI and STEMI), one-way ANOVA was applied for normally distributed variables, followed by the post hoc Tukey test to identify significant group differences. For non-normally distributed variables, the Kruskal–Wallis test was used, with post hoc pairwise comparisons adjusted using the Bonferroni correction. Chi-square or Fisher’s exact tests were employed for categorical data. Correlations between Nt-proBNP, TM, H-FABP, PTX-3, Gal-3 and other clinical parameters were assessed using Spearman’s correlation analysis. Receiver operating characteristic (ROC) curves were constructed to evaluate the diagnostic performance of novel biomarkers in differentiating among groups. The area under the curve (AUC) with 95% confidence intervals (CIs) was reported. Optimal cut-off values were determined using the Youden index, and sensitivity and specificity were calculated. A *p*-value < 0.05 was considered statistically significant for all tests.

## 3. Results

The proportion of male participants was 53.3% in the NSTEMI, 55.6% in the STEMI groups and 42.2% in the control group, although the difference was not statistically significant (*p* = 0.159). Smoking, alcohol use and the presence of comorbidities such as diabetes mellitus (DM), hypertension (HT) and cardiovascular complications were significantly more prevalent in the NSTEMI and STEMI groups than in the control group (all *p* < 0.001) (Table 1).

The mean ± SD age did not differ significantly among the control (58.22 ± 8.11 years), NSTEMI (59.36 ± 7.74 years) and STEMI (58.81 ± 9.27 years) groups (*p* = 0.664). The control group had significantly lower BMI (24.03 ± 1.39 kg/m^2^) than the NSTEMI (26.9 ± 3.76 kg/m^2^) and STEMI (27.18 ± 3.36 kg/m^2^) groups (*p* < 0.001). Systolic blood pressure (SBP) was different significantly across groups: 125 mmHg (120–129) in controls, 135 mmHg (130–155) in NSTEMI and 145 mmHg (144–155) in STEMI (*p* < 0.001). Total cholesterol levels were significantly lower in the control group (median 165.5 mg/dL (150–184)) compared to the NSTEMI (median 196 mg/dL (180–222)) and STEMI groups (median 235 mg/dL (184–243); *p* < 0.001). LDL cholesterol levels were significantly higher in the STEMI group (median 150 mg/dL (124–182)) compared to the NSTEMI (median 100 mg/dL (85–112)) and control groups (median 91.5 mg/dL (83–102); *p* < 0.001). HDL cholesterol levels showed significant differences across all three groups, with the highest levels in the control group (median 45 mg/dL (36–49)), followed by the NSTEMI (median 39 mg/dL (34–45)) and STEMI groups (median 36 mg/dL (30–40); *p* < 0.001). Triglyceride levels were significantly lower in the control group (median 96 mg/dL (78–114)) compared to the NSTEMI (median 120 mg/dL (79–139)) and STEMI groups (median 119 mg/dL (96–150); *p* < 0.001) (Table 2).

Fibrinogen levels were significantly lower in the control group (median 2.89 g/L (2.44–3.4)) compared to the NSTEMI (median 5.2 g/L (4.2–5.57)) and STEMI groups (median 4.99 g/L (4.63–5.9); *p* < 0.001). CRP levels were also significantly lower in the control group (median 2.7 mg/L (2.51–3.23)) compared to the NSTEMI (median 17.55 mg/L (9.48–22.6)) and STEMI groups (median 19.75 mg/L (11.97–32.02); *p* < 0.001) (Table 2).

Hs-TnI levels demonstrated significant differences across all three groups, with medians of 0.05 ng/mL (0.04–0.07) in controls, 0.21 ng/mL (0.07–2.13) in NSTEMI and 38.77 ng/mL (24.65–45.36) in STEMI (*p* < 0.001). CK-MB levels also differed significantly among the groups, with medians of 14.4 ng/mL (11.55–18.6) in controls, 87.64 ng/mL (82.03–92.07) in NSTEMI and 257 ng/mL (200–395) in STEMI (*p* < 0.001) (Table 2).

**Figure 1:**
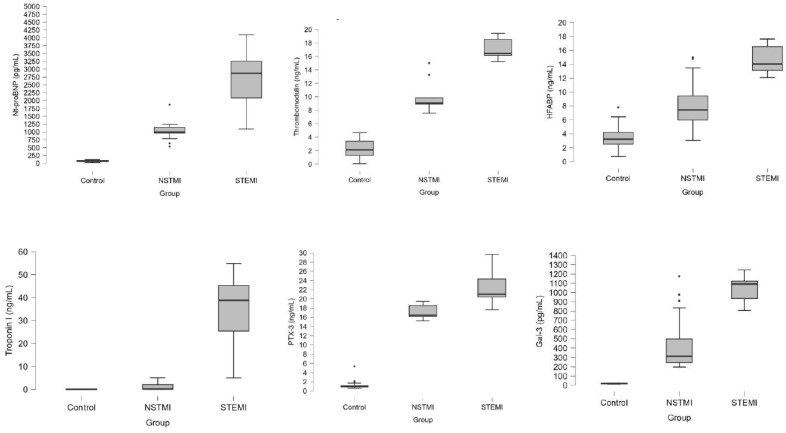
Box-plot graphs of hs-Tn I, NT-proBNP, TM, H-FABP, PTX-3 and Gal-3 levels across control, NSTEMI and STEMI groups. Note: The dots in the figures are extreme values.

NT-proBNP levels showed significant differences across all groups, with medians of 74 pg/mL (59–96) in controls, 998 pg/mL (963–1152) in NSTEMI and 2869 pg/mL (2086–3254) in STEMI (*p* < 0.001). TM levels were also significantly different, with medians of 2.12 ng/mL (1.23–3.45) in controls, 9.06 ng/mL (8.92–9.86) in NSTEMI and 16.45 ng/mL (16.15–18.56) in STEMI (*p* < 0.001). H-FABP levels were significantly higher in the STEMI group (median 14.04 ng/mL (13.12–16.54)) compared to the NSTEMI (median 7.42 ng/mL (5.9–9.45)) and control groups (median 3.23 ng/mL (2.51–4.23); *p* < 0.001). PTX-3 levels differed significantly among all groups, with medians of 0.98 ng/mL (0.85–1.2) in controls, 16.45 ng/mL (16.15–18.56) in NSTEMI and 21.03 ng/mL (20.42–24.32) in STEMI (*p* < 0.001). Similarly, Gal-3 levels showed significant differences, with medians of 19.23 pg/mL (16.23–23.1) in controls, 313.8 pg/mL (245.65–498.45) in NSTEMI and 1092.78 pg/mL (936.75–1124.02) in STEMI (*p* < 0.001) (Table 2, Figure 1).

NT-proBNP levels showed strong positive correlations with TM (r = 0.915, *p* < 0.001), H-FABP (r = 0.844, *p* < 0.001), PTX-3 (r = 0.910, *p* < 0.001) and Gal-3 (r = 0.921, *p* < 0.001). TM levels were strongly correlated with H-FABP (r = 0.854, *p* < 0.001), PTX-3 (r = 0.914, *p* < 0.001) and Gal-3 (r = 0.919, *p* < 0.001). H-FABP exhibited significant positive correlations with PTX-3 (r = 0.864, *p* < 0.001) and Gal-3 (r = 0.854, *p* < 0.001). PTX-3 levels were positively correlated with Gal-3 (r = 0.905, *p* < 0.001). When the correlation between NT-proBNP, TM, H-FABP and Gal-3 was evaluated on a group basis, no correlation was observed in the control and NSTEMI groups, whereas strong and significant correlations were observed in the STEMI group as in all groups (Table 3). 

ROC analysis demonstrated that in distinguishing STEMI from controls, all biomarkers achieved perfect discrimination (AUC: 1.00) with optimal cut-off values, as follows: NT-proBNP (cut-off: 125 pg/mL, sensitivity: 100%, specificity: 100%), TM (cut-off: 5 ng/mL, sensitivity: 100%, specificity: 100%), H-FABP (cut-off: 10 ng/mL, sensitivity: 100%, specificity: 100%), PTX-3 (cut-off: 5.5 ng/mL, sensitivity: 100%, specificity: 100%), Gal-3 (cut-off: 30 pg/mL, sensitivity: 100%, specificity: 100%), hs-TnI (cut-off: 0.10 ng/mL, sensitivity: 100%, specificity: 100%) and CK-MB (cut-off: 30 U/L, sensitivity: 100%, specificity: 100%).

In differentiating STEMI from NSTEMI, three biomarkers demonstrated exceptional diagnostic accuracy, TM (cut-off: 15 ng/mL, sensitivity: 100%, specificity: 100%), hs-TnI (cut-off: 5 ng/mL, sensitivity: 100%, specificity: 98.9%) and CK-MB (cut-off: 110 U/L, sensitivity: 100%, specificity: 100%), all achieving an AUC of 1.00. Among other biomarkers, Gal-3 (AUC: 0.981) and NT-proBNP (AUC: 0.966) also demonstrated high diagnostic accuracy, with Gal-3 achieving 100% sensitivity and 94.44% specificity at 600 pg/mL cut-off.

In distinguishing NSTEMI from controls, CK-MB demonstrated excellent diagnostic accuracy (AUC: 1.00) with 100% sensitivity and specificity at a cut-off value of 30 U/L, while hs-TnI showed relatively lower performance (AUC: 0.779, 95% CI: 0.699–0.860) with 71.1% sensitivity and 100% specificity at a cut-off value of 0.10 ng/mL. The remaining biomarkers maintained high diagnostic accuracy with their respective cut-off values (Table 4).

## 4. Discussion

Early diagnosis and risk assessment in ACS with STEMI and NSTEMI are important in determining both appropriate treatment and prognosis. In this study, TM, H-FABP, PTX-3 and Gal-3 levels were significantly higher in STEMI and NSTEMI patients compared to the control group. In STEMI, NT-proBNP levels were strongly correlated with TM, HFABP, PTX-3 and Gal-3. TM levels were strongly correlated with H-FABP, PTX-3 and Gal-3. H-FABP exhibited significant positive correlations with PTX-3 and Gal-3. PTX-3 levels were positively correlated with Gal-3. According to ROC analysis, serum NT-proBNP, TM, H-FABP, PTX-3 and Gal-3 levels have diagnostic accuracy in distinguishing STEMI from control and NSTEMI groups. Vascular inflammation plays an important role in the pathophysiology of STEMI and NSTEMI. We also believe in the findings from current studies indicating that TnI, CK-MB, NT-proBNP, TM, H-FABP, PTX-3 and Gal-3 as panel indicators will be useful.

TM is an important modulator of intravascular coagulation and inflammation. It is found on many endothelial cells and tumor cells. In the present study, TM levels were significantly different in STEMI compared to control and NSTEMI groups. The highest was in the STEMI group. TM serves as a cofactor for thrombin, converting it from a pro-coagulant to an anticoagulant enzyme by activating protein C. This pathway has implications in the pathogenesis of MI due to its role in thrombosis and vascular integrity. in a mouse model of AMI, the same phenomena as that found in human samples were observed by immunohistochemistry of TM. Immunostaining of TM, as a marker for endothelial injury or myocardial remodeling, may be useful for supplementing conventional staining techniques in the diagnosis of ischemic heart disease in forensic pathology. Kondo et al. [8] reported that TM plays a dual role in the pathophysiology of MI, acting as both a marker of endothelial damage and a potential therapeutic target. The thrombomodulin (THBD) gene, which encodes TM, a critical endothelial glycoprotein, has garnered attention as a promising diagnostic marker for AMI. The role of the THBD gene and its product in maintaining vascular integrity, anticoagulation and inflammation modulation directly links it to the pathophysiology of AMI [5,24]. TM levels in hemostasis disorders of CAD are controversial. TM was lower in NSTEMI patients compared to unstable angina (UA) patients and controls [25]. This paradox may be due to the presence of cardiovascular risk factors such as smoking, diabetes mellitus, arterial hypertension, dyslipidemia, chronic kidney disease (CKD) and peripheral arterial disease (PAD). Elevated TM levels may complement traditional biomarkers such as troponins, providing additional insights into vascular and endothelial contributions to AMI. TM levels could also differentiate AMI from other forms of myocardial injury.

H-FABP is a low-molecular-weight cytoplasmic protein primarily found in cardiac myocytes. It plays a crucial role in intracellular fatty acid transport and metabolism. H-FABP is released within 30 min to 3 h after myocardial injury, making it one of the earliest biomarkers detectable in the blood. It peaks at around 6–8 h and returns to baseline within 24 h, providing a narrow diagnostic window [26]. However, rapid clearance from circulation limits its utility in late presentations of MI. In the present study, H-FABP levels were significantly higher in the STEMI group compared to the NSTEMI and control groups. H-FABP can detect myocardial injury earlier than traditional biomarkers such as troponins, particularly in the first few hours’ post-injury. It is especially valuable in patients presenting with symptoms of MI but with initially normal troponin levels. While H-FABP is found in other tissues (e.g., skeletal muscle), its concentration is significantly higher in cardiac myocytes, enhancing its specificity for cardiac injury when used in conjunction with other biomarkers like troponins. Due to its rapid release into the bloodstream following myocardial injury, H-FABP has emerged as a promising early biomarker for detecting MI and other cardiac injuries [27,28,29]. Although it has high specificity and sensitivity in differentiating STEMI from NSTEMI in our study, H-FABP is not typically used as an individual biomarker. However, it should be used in combination with cardiac troponins to improve diagnostic accuracy and early detection of MI. H-FABP is a highly sensitive and early biomarker for myocardial injury, with the potential to complement troponin assays in ACS diagnosis. Its rapid release and prognostic implications make it a valuable tool in the early stages of MI evaluation. Its further integration into diagnostic protocols and combined use with other markers could enhance its clinical utility.

PTX-3 is an acute-phase glycoprotein that is produced by a variety of cell types, including vascular endothelial cells, smooth muscle cells, macrophages and cardiac myocytes, in response to inflammatory stimuli. PTX-3 has been increasingly studied as a biomarker of CVD, including myocardial injury, due to its role in inflammation, endothelial dysfunction and tissue damage [30,31,32,33]. In the present study, PTX-3 levels were significantly different between all groups, with the highest levels in the STEMI group. Consistent with the results of Befekadu et al. [30], a weak correlation was found between PTX3 and troponin I in our study. PTX3 is released rapidly within hours after ischemic or inflammatory injury, including MI. Unlike C-reactive protein (CRP), which is produced primarily by the liver, PTX3 is synthesized locally at the site of vascular or myocardial injury, making it a more direct marker of tissue inflammation [31]. Befekadu et al. [30] reported PTX3 levels being high in the acute samples, while the peak for CRP came 1–3 days after percutaneous coronary intervention (PCI). Elevated PTX-3 levels correlate with the extent and severity of myocardial ischemia and infarction. PTX-3 plays a role in the regulation of complement activation, preventing excessive immune-mediated tissue damage in ischemic areas. PTX3 levels rise within 2–3 h of myocardial injury and peak earlier than traditional biomarkers such as troponins. It may help in the early detection of myocardial injury, particularly in patients presenting during the hyperacute phase of MI. PTX-3 levels rise quickly, providing early insights into myocardial injury. PTX-3 reflects local inflammatory and ischemic responses rather than systemic inflammation. Assays for PTX-3 measurements are not yet widely standardized, limiting its clinical use. PTX-3 levels may be elevated in other inflammatory or ischemic conditions, potentially confounding its specificity for myocardial injury. PTX-3 is unlikely to replace traditional markers like troponins but can complement them in diagnostic and prognostic systems [34]. PTX-3 is a promising biomarker for myocardial injury, reflecting local inflammation, endothelial dysfunction and the extent of cardiac damage. Its rapid rise after myocardial ischemia and strong prognostic value makes it a valuable tool for early detection and risk stratification [35]. However, further research is required to standardize its clinical application and integrate it into routine cardiovascular diagnostics. PTX3 as an inflammatory marker showed higher levels in patients with MI, especially in STEMI. Therefore, combined evaluation of troponin I and PTX3 levels would be associated with more accuracy in diagnosis of MI [36].

Gal-3 are a family of beta-galactoside-binding proteins involved in various cellular processes, including inflammation, apoptosis and tissue remodeling. Among the galectin family, Gal-3 has been most extensively studied in the context of cardiovascular diseases, including MI [37]. In the present study, as in other biomarkers, Gal-3 levels were found to be significantly higher in STEMI compared to NSTEMI and controls. Its role as both a biomarker and a mediator of myocardial injury highlights its significance in the diagnosis, prognosis and potential treatment of MI [37]. Gal-3 is secreted by activated macrophages and plays a critical role in the inflammatory response following MI. It promotes the recruitment and activation of immune cells at the site of injury, contributing to inflammation and tissue damage. Gal-3 is directly involved in cardiac fibrosis by stimulating fibroblast activation and collagen deposition. After MI, its overexpression can exacerbate ventricular remodeling, leading to impaired cardiac function and heart failure [38]. Elevated Gal-3 levels in the blood can indicate ongoing myocardial injury and inflammatory processes. It may serve as an adjunct to traditional biomarkers like cardiac troponins for a more comprehensive evaluation of MI. High circulating levels of Gal-3 are associated with worse outcomes in MI patients, including increased mortality, heart failure development and adverse ventricular remodeling. Gal-3 provides prognostic information beyond established markers, aiding in risk stratification [39,40,41]. Vucic et al. [42] reported that Gal-3 plasma concentrations in arterial and venous blood and central and peripheral blood on day one of AMI increased in patients who developed major adverse cardiovascular event at six months follow-up. Galectin-3 is also elevated in non-cardiac conditions, such as chronic kidney disease, cancer and systemic inflammation, potentially reducing its specificity for MI. Unlike troponins, Gal-3 levels do not rise immediately after myocardial injury, limiting its use as an early diagnostic marker [20]. Variation in assay methods and cutoff values poses challenges for clinical implementation. Using Gal-3 alongside troponins and natriuretic peptides may enhance diagnostic and prognostic accuracy. Further research is needed to evaluate Gal-3 inhibitors as potential therapies to reduce fibrosis and adverse remodeling after MI. Although Gal-3 has high specificity and sensitivity in differentiating STEMI and NSTEMI, investigating genetic variations and regulatory pathways of Gal-3 could provide deeper insights into its role in MI. Gal-3 is a multifunctional protein with significant roles in the pathophysiology of MI, particularly in inflammation, fibrosis and remodeling. As a biomarker, it offers both diagnostic and prognostic value, especially when combined with other markers. Its involvement in pathological processes also makes it a promising therapeutic target for improving outcomes in MI patients. In a 2024 study [43], despite the well-known cardiovascular benefits observed in the general population with T2DM and in other patient groups regardless of diabetes status, current evidence does not support the use of sodium-glucose cotransporter-2 (SGLT2) inhibitors in the context of ACS. The identification of high-risk NSTEMI patients is important, as they may require an individualized approach that can substantially overlap with current STEMI recommendations, and their mortality remains high if their management is delayed [44]. Definitive answers to this intriguing research question, which could potentially expand the therapeutic indications of this novel drug class, require large-scale, well-designed therapy [43].

The current guidelines clearly change the recommendation for the pretreatment of STEMI patients, which was level IA and is now only IIbB: “Pre-treatment with a P2Y12 receptor inhibitor may be considered in patients undergoing a primary PCI strategy.” The change was made due to a revised appraisal of the current evidence, which does not clearly support pre-treatment in randomized trials [44], but more so in older observational data [45], which are nevertheless convincing related to the pathomechanism [46,47].

In differentiating STEMI from NSTEMI, three biomarkers demonstrated exceptional diagnostic accuracy—TM (cut-off: 15 ng/mL, sensitivity: 100%, specificity: 100%), hs-TnI (cut-off: 5 ng/mL, sensitivity: 100%, specificity: 98.9%) and CK-MB (cut-off: 110 U/L, sensitivity: 100%, specificity: 100%), all achieving an AUC of 1.00. Among other biomarkers, Gal-3 (AUC: 0.981) and NT-proBNP (AUC: 0.966) also demonstrated high diagnostic accuracy, with Gal-3 achieving 100% sensitivity and 94.44% specificity at 600 pg/mL cut-off. These cut-off values may contribute to the differentiation of STEMI from NSTEMI in ACS.The diagnosis and management of STEMI and NSTEMI rely on integrating clinical assessment, electrocardiographic findings and biomarker analysis. Biomarkers play an essential role in confirming myocardial injury, assessing severity, stratifying risk and guiding treatment. Advances in biomarker research have provided a deeper understanding of the pathophysiology of MI and enhanced clinical decision making. TnI, the gold standard biomarker for myocardial necrosis, is essential for diagnosing both STEMI and NSTEMI. CK-MB is useful for early detection and identifying reinfarction due to its shorter half-life. NT-proBNP reflects ventricular wall stress, aiding in risk stratification and the assessment of heart failure co-occurrence. 

## 5. Conclusions

TM, a marker of endothelial dysfunction, provides prognostic insights into vascular injury and thrombotic risk. H-FABP, an early marker of myocardial injury, complements troponins in early presentations. PTX-3 indicates localized inflammation, contributing to risk stratification and predicting adverse outcomes. Gal-3, a marker of fibrosis and adverse ventricular remodeling, is crucial for predicting long-term outcomes and heart failure progression. In STEMI, immediate recognition via ECG and rapid elevation of TnI or CK-MB confirms the diagnosis, and biomarkers like PTX-3 and Gal-3 may provide additional prognostic value. In NSTEMI, diagnosis often relies on TnI and NT-proBNP levels, especially in the absence of ST-elevation on ECG. Early markers like H-FABP can aid in detecting myocardial injury before troponin levels rise. Emerging biomarkers such as PTX-3, Gal-3 and TM hold promise for enhancing the diagnostic and prognostic toolkit in AMI. Their integration into clinical practice could lead to personalized treatment strategies and improved outcomes. A comprehensive cardiac biomarker panel enhances diagnostic accuracy and risk stratification, particularly when distinguishing between STEMI and NSTEMI. The biomarkers TnI, CK-MB, NT-proBNP, TM, H-FABP, PTX-3 and Gal-3 offer complementary information when used together as a panel. Further research and validation are essential to establish standardized protocols for their widespread use. More detailed studies are needed to confirm the findings in larger, multicenter cohorts, and further research is needed to investigate cost-effectiveness.

## Figures and Tables

**Table 1:** Demographic characteristics and comorbidities of control, NSTEMI and STEMI groups.

	Group						
	Control		NSTMI		STEMI		
	*n*	%	*n*	%	*n*	%	*p* Value
Sex							
Female	52	57.8%	42	46.7%	40	44.4%	0.159
Male	38	42.2%	48	53.3%	50	55.6%	
Smoking	0	0.0%	22	24.4%	60	66.7%	<0.001
Alcohol	0	0.0%	17	18.9%	17	18.9%	<0.001
Co-morbidity	0	0.0%	46	51.1%	55	61.1%	<0.001
Diabetes mellitus (DM)	0	0.0%	19	21.1%	45	50.0%	<0.001
Hypertension (HT)	0	0.0%	44	48.9%	49	54.4%	<0.001
Cardiovascular complications	0	0.0%	9	10.0%	12	13.3%	0.002

Chi-square test was applied.

**Table 2:** Comparison of clinical, hemodynamic and biochemical parameters across study groups.

	Control (*n*:90)	NSTEMI (*n*:90)	STEMI (*n*:90)	*p* Value
Age (Year)	58.22 ± 8.11	59.36 ± 7.74	58.81 ± 9.27	0.664
BMI (kg/m^2^)	24.03 ± 1.39 ^a^	26.9 ± 3.76 ^b^	27.18 ± 3.36 ^b^	<0.001
SBP (mmHg)	125 (120–129) ^a^	135 (130–155) ^b^	145 (144–155) ^c^	<0.001
DBP (mmHg)	74.5 (68–75) ^a^	83.5 (78–86) ^b^	82.5 (77–95)	<0.001
Pulse (bpm)	78 (72–80) ^a^	75 (66–80) ^a^	80 (77–88) ^b^	<0.001
Fasting blood glucose (FBG) (mg/dL)	93 (86–96) ^a^	91.5 (85–100) ^a^	109.5 (91–141) ^b^	<0.001
HbA1c (%)	5.2 (5.1–5.5) ^a^	5.7 (5.1–5.8) ^b^	6 (5.6–7.9) ^c^	<0.001
T. cholesterol (mg/dL)	165.5 (150–184) ^a^	196 (180–222) ^b^	235 (184–243) ^b^	<0.001
LDL (mg/dL)	91.5 (83–102) ^a^	100 (85–112) ^a^	150 (124–182) ^b^	<0.001
HDL (mg/dL)	45 (36–49) ^a^	39 (34–45) ^b^	36 (30–40) ^c^	<0.001
Triglycerid (mg/dL)	96 (78–114) ^a^	120 (79–139) ^b^	119 (96–150) ^b^	<0.001
Fibrinogen (g/L)	2.89 (2.44–3.4) ^a^	5.2 (4.2–5.57) ^b^	4.99 (4.63–5.9) ^b^	<0.001
High-sensitivity troponin (hs-TnI) (ng/mL)	0.05 (0.04–0.07) ^a^	0.21 (0.07–2.13) ^b^	38.77 (24.65–45.36) ^c^	<0.001
Creatin kinase -MB (CK-MB) (ng/mL)	14.4 (11.55–18.6) ^a^	87.64 (82.03–92.07) ^b^	257 (200–395) ^c^	<0.001
C-Reactive Protein (CRP) (mg/L)	2.7 (2.51–3.23) ^a^	17.55 (9.48–22.6) ^b^	19.75 (11.97–32.02) ^b^	<0.001
NT-proBNP (pg/mL)	74 (59–96) ^a^	998 (963–1152) ^b^	2869 (2086–3254) ^c^	<0.001
Thrombomodulin (TM) (ng/mL)	2.12 (1.23–3.45) ^a^	9.06 (8.92–9.86) ^b^	16.45 (16.15–18.56) ^c^	<0.001
Heart-type fatty-acid-binding protein (H-FABP) (ng/mL)	3.23 (2.51–4.23) ^a^	7.42 (5.9–9.45) ^b^	14.04 (13.12–16.54) ^c^	<0.001
Pentraxin-3 (PTX-3) (ng/mL)	0.98 (0.85–1.2) ^a^	16.45 (16.15–18.56) ^b^	21.03 (20.42–24.32) ^c^	<0.001
Galectin-3 (Gal-3) (pg/mL)	19.23 (16.23–23.1) ^a^	313.8 (245.65–498.45) ^b^	1092.78 (936.75–1124.02) ^c^	<0.001

Data were presented as mean + SD or median (IQR). One-way ANOVA was used for age, and BMI and Kruskal–Wallis test was used for other comparisons. After one-way ANOVA, post hoc Tukey test was used to find the groups in which the difference originated, and adjusted *p* values were used after Kruskal–Wallis test. Different superscript letters (a,b,c) indicate groups with significant differences.

**Table 3:** Correlation coefficients between NT-proBNP, thrombomodulin, H-FABP, PTX-3, galectin-3 and clinical parameters in study groups.

		Control					NSTEMI					STEMI				
		NT-proBNP	TM	H-FABP	PTX-3	Gal-3	NT-proBNP	TM	H-FABP	PTX-3	Gal-3	Nt-proBNP	TM	H-FABP	PTX-3	Gal-3
NT-proBNP (pg/mL)	r		−0.137	−0.081	−0.067	0.134		0.053	−0.039	0.081	0.042		0.961	0.891	0.915	0.979
	*p*		0.196	0.446	0.532	0.209		0.621	0.716	0.450	0.696		<0.001	<0.001	<0.001	<0.001
TM (ng/mL)	r			−0.153	0.076	−0.074			−0.005	0.044	0.048			0.861	0.870	0.950
	*p*			0.150	0.478	0.490			0.963	0.680	0.652			<0.001	<0.001	<0.001
H-FABP (ng/mL)	r				0.048	−0.031				0.151	0.082				0.866	0.892
	*p*				0.651	0.769				0.156	0.442				<0.001	<0.001
PTX-3 (ng/mL)	r					−0.128					0.065					0.950
	*p*					0.228					0.541					<0.001
hs-Tn I (ng/mL)	r	−0.061	−0.133	−0.110	0.025	−0.017	−0.056	0.006	−0.052	0.029	−0.084	0.010	−0.019	−0.017	−0.062	−0.026
	*p*	0.569	0.211	0.301	0.816	0.873	0.599	0.954	0.623	0.789	0.433	0.925	0.857	0.875	0.563	0.811

**Table 4:** Diagnostic performance of novel biomarkers (NT-proBNP, thrombomodulin, H-FABP, PTX-3, Galectin-3) in distinguishing among groups.

	Variable(s)	AUC	95% CI for AUC	*p* Value	Cut-Off	Sensitivity	Specificity
C vs. ST	Nt_proBNP (pg/mL)	1.000	1–1	<0.001	125	100%	100%
	Thrombomodulin (ng/mL)	1.000	1–1	<0.001	5	100%	100%
	HFABP (ng/mL)	1.000	1–1	<0.001	10	100%	100%
	PTX-3 (ng/mL)	1.000	1–1	<0.001	5.5	100%	100%
	Gal-3 (pg/mL)	1.000	1–1	<0.001	30	100%	100%
	Troponin I	1.000	1–1	<0.001	0.10	100%	100%
	CK-MB	1.000	1–1	<0.001	30	100%	100%
C vs. NST	Nt_proBNP (pg/mL)	1.000	1–1	<0.001	125	100%	100%
	Thrombomodulin (ng/mL)	1.000	1–1	<0.001	5	100%	100%
	HFABP (ng/mL)	0.922	0.884–0.959	<0.001	4.75	93.3%	78.9%
	PTX-3 (ng/mL)	1.000	1–1	<0.001	5.5	100%	100%
	Gal-3 (pg/mL)	1.000	1–1	<0.001	30	100%	100%
	Troponin I	0.779	0.699–0.860	<0.001	0.10	71.1%	100%
	CK-MB	1.000	1–1	<0.001	30	100%	100%
NST vs. ST	Nt_proBNP (pg/mL)	0.966	0.944–0.989	<0.001	1250	88.89%	98.89%
	Thrombomodulin (ng/mL)	1.000	1–1	<0.001	15	100%	100%
	HFABP (ng/mL)	0.926	0.885–0.66	<0.001	10	100.00%	85.56%
	PTX-3 (ng/mL)	0.953	0.924–0.981	<0.001	19.5	86.67%	100.00%
	Gal-3 (pg/mL)	0.981	0.961–1	<0.001	600	100.00%	94.44%
	Troponin I	1.000	0.999–1	<0.001	5	100%	98.9%
	CK-MB	1.000	1–1	<0.001	110	100%	100%

## Data Availability

The datasets used and analyzed in this study are available from the corresponding authors upon reasonable request.
